# Clinical and patient-reported outcomes in women offered oncoplastic breast-conserving surgery as an alternative to mastectomy: ANTHEM multicentre prospective cohort study

**DOI:** 10.1093/bjs/znae306

**Published:** 2024-12-24

**Authors:** Charlotte Davies, Leigh Johnson, Carmel Conefrey, Nicola Mills, Patricia Fairbrother, Chris Holcombe, Lisa Whisker, William Hollingworth, Joanna Skillman, Paul White, Douglas Macmillan, Charles Comins, Shelley Potter

**Affiliations:** Bristol Surgical and Perioperative Care Complex Intervention Collaboration, Translational Health Sciences, Bristol Medical School, University of Bristol, Learning and Research Building, Southmead Hospital, Bristol, UK; Population Health Sciences, Bristol Medical School, Bristol, UK; Population Health Sciences, Bristol Medical School, Bristol, UK; Population Health Sciences, Bristol Medical School, Bristol, UK; Independent Cancer Patients Voice (ICPV), London, UK; Linda McCartney Centre, Royal Liverpool and Broadgreen University Hospital, Liverpool, UK; Nottingham Breast Institute, Nottingham University Hospitals NHS Trust, Nottingham, UK; Population Health Sciences, Bristol Medical School, Bristol, UK; Department of Plastic Surgery, University Hospitals Coventry and Warwickshire NHS Trust, Coventry, UK; Applied Statistics Group, University of the West of England, Bristol, UK; Nottingham Breast Institute, Nottingham University Hospitals NHS Trust, Nottingham, UK; Bristol Haematology and Oncology Centre, University Hospitals Bristol Foundation NHS Trust, Bristol, UK; Bristol Surgical and Perioperative Care Complex Intervention Collaboration, Translational Health Sciences, Bristol Medical School, University of Bristol, Learning and Research Building, Southmead Hospital, Bristol, UK; Bristol Breast Care Centre, Southmead Hospital, Bristol, UK

## Abstract

**Background:**

Oncoplastic breast-conserving surgery may be a better option than mastectomy, but high-quality comparative evidence is lacking. The aim of the ANTHEM study (ISRCTN18238549) was to explore clinical and patient-reported outcomes in a multicentre cohort of women offered oncoplastic breast-conserving surgery as an alternative to mastectomy with or without immediate breast reconstruction.

**Methods:**

Women with invasive/pre-invasive breast cancer who were offered oncoplastic breast-conserving surgery with volume replacement or displacement techniques to avoid mastectomy were recruited prospectively. Demographic, operative, oncological, and 3- and 12-month complication data were collected. The proportion of women choosing oncoplastic breast-conserving surgery and the proportion in whom breast conservation was successful were calculated. Participants completed the validated BREAST-Q questionnaire at baseline, 3 months after surgery, and 12 months after surgery. Questionnaires were scored according to the developers’ instructions and scores for each group were compared over time.

**Results:**

In total, 362 women from 32 UK breast units participated, of whom 294 (81.2%) chose oncoplastic breast-conserving surgery. Of the oncoplastic breast-conserving surgery patients in whom postoperative margin status was reported, 210 of 255 (82.4%) had clear margins after initial surgery and only 10 (3.9%) required completion mastectomy. Major complications were significantly more likely after immediate breast reconstruction. Women having oncoplastic breast-conserving surgery with volume displacement techniques reported significant improvements in baseline ‘satisfaction with breasts’ and ‘psychosocial well-being’ scores at 3 and 12 months, but both oncoplastic breast-conserving surgery groups reported significant decreases in ‘physical well-being: chest’ at 3 and 12 months.

**Conclusion:**

Oncoplastic breast-conserving surgery allows greater than 95% of women to avoid mastectomy, with lower major complication rates than immediate breast reconstruction, and may improve satisfaction with outcome. Oncoplastic breast-conserving surgery should be offered as an alternative to mastectomy in all women in whom it is technically feasible.

## Teaser

Oncoplastic breast-conserving surgery may allow many women to avoid mastectomy, but there are limited data regarding the acceptability of this approach or the outcomes of surgery in a cohort of women offered both options. In this multicentre prospective cohort study, over 80% of women offered oncoplastic breast-conserving surgery to avoid mastectomy chose this option and over 90% had successful breast conservation. Women having oncoplastic breast-conserving surgery experienced lower major complication rates and reported better satisfaction than those having mastectomy with or without breast reconstruction, suggesting oncoplastic breast-conserving surgery may be a better option for many women.

## Introduction

Over 56 000 women are diagnosed with breast cancer every year in the UK^1^ and, despite improvements in treatment, approximately 40% will undergo mastectomy^2^, which may profoundly impact their quality of life^3^. Women describe mastectomy as a ‘mutilating’ and ‘disfiguring’ procedure that most would prefer to avoid if safe alternatives were available^4^.

Breast-conserving surgery (BCS) with radiotherapy has long been established as an oncologically safe alternative to mastectomy in landmark randomized trials^5,6^, but recent meta-analyses of contemporaneous observational cohorts suggest that BCS may offer improved long-term survival compared with mastectomy^7–9^.

There is therefore a drive to optimize BCS and avoid mastectomy, but standard BCS techniques are limited by tumour size and excision of more than 10–20% of breast volume can lead to unacceptable cosmetic outcomes^10,11^ and poor quality of life^12^. Oncoplastic breast-conserving surgery (OPBCS) techniques, which combine removal of the breast cancer with plastic surgical volume displacement^13^ or replacement^14^ techniques to reduce, lift, or reconstruct the breast mound, extend the indications for BCS by allowing large resections whilst maintaining an acceptable breast form^11^. OPBCS is considered oncologically safe, even in patients with extensive or multicentric disease^15–19^, and its effective use has been shown to reduce mastectomy rates^20–22^. Additional benefits of OPBCS compared with mastectomy with or without immediate breast reconstruction (IBR) may include: fewer postoperative complications, even in high risk groups^23^; better quality of life^24,25^; and improved cost-effectiveness^26^.

While there is an increasingly compelling argument that OPBCS is a better option than mastectomy with or without IBR, there are few comparative studies^27,28^ and existing evidence to support the benefits of OPBCS, particularly the more recently introduced volume replacement techniques, is limited^19,22–25,27,28^. The best current evidence for the chest wall perforator flap (CWPF) procedure comes from a recently published international multicentre cohort of 603 patients, two-thirds of whom were offered the procedure to avoid mastectomy^22^. The completion mastectomy rate in this study was 1.5% and the overall complication rate was low, supporting the benefits of the technique. Of the comparative studies, most are single-centre studies^17,24,25^, few have included patient-reported outcomes^24,25^, and it is often unclear whether the groups having OPBCS and mastectomy are directly comparable. Well-designed, large-scale studies evaluating the outcomes of OPBCS as an alternative to mastectomy are needed, but randomized trials are not feasible due to patient and surgeon preference^29^. Preliminary work is therefore essential to determine the acceptability of OPBCS as an alternative to mastectomy with or without IBR and women’s preferences for different surgical options, and to explore the clinical and patient-reported outcomes of surgery in a cohort of women explicitly offered the choice of both procedures.

The aim of the multicentre prospective cohort phase of the ANTHEM study (ISRCTN18238549) was to explore the feasibility of undertaking a large-scale prospective study comparing the clinical and patient-reported outcomes of OPBCS as an alternative to mastectomy with or without IBR.

## Methods

The ANTHEM study was a mixed-methods feasibility study, with four phases^30^. This paper reports phase two, the multicentre prospective cohort study. The national practice survey^31^ and qualitative interview study^32^ have been reported elsewhere.

All UK breast and plastic surgical centres performing level two OPBCS, defined as offering complex oncoplastic volume displacement and/or replacement techniques, were invited to participate in the study.

### Study design and participants

Full details of the study design and methods have been reported elsewhere^30^. In brief, women over the age of 18 years with newly diagnosed primary invasive breast cancer or ductal carcinoma *in situ* (‘DCIS’) and offered level two OPBCS^11^ to allow them to avoid mastectomy were eligible to participate in the study. Either volume displacement (therapeutic mammaplasty (TM)) or replacement (CWPF) techniques could be offered as an alternative to mastectomy with or without IBR. Women not offered a choice of OPBCS *versus* mastectomy, those who could be managed with simple BCS/level one oncoplastic techniques, and those offered OPBCS for reasons other than to avoid mastectomy (for example to avoid impact of radiotherapy in large breasts) were excluded.

Full ethical approval was obtained from Wales Research Ethics Committee 6 (reference number 20/WA/0225) and the study was prospectively registered in the ISRCTN registry before recruitment was commenced (trial registration number ISRCTN18238549).

### Procedures

Women considered suitable for OPBCS as an alternative to mastectomy were identified via local multidisciplinary team (MDT) meetings. Women were assessed by their operating surgeon and offered OPBCS and mastectomy with or without IBR as appropriate. Women offered OPBCS specifically to avoid mastectomy as determined by their operating surgeon after consideration of the total extent of disease in relation to the breast size and given a choice of procedures by their surgical team were eligible to participate. Information provision was as per the clinical teams’ standard local practice and it was not mandated that the options should be offered equally. They were given a participant information sheet outlining the study and followed up by local research teams. Those electing to participate provided written informed consent.

All patients were given an operation date as per local unit policy and simple demographic and preoperative planning data were collected using standardized case report forms (‘CRFs’) via the online REDCap database^33^.

Participants underwent their procedure of choice and were followed up according to local practice. Adjuvant treatment recommendations were made after local MDT discussion as per local guidelines.

Oncological data at 3 months and complications at 3 and 12 months were collected by clinical or case-note review according to local policy. No additional clinic visits were required for the study. Complications were defined a priori using standardized definitions used in other oncoplastic and reconstructive surgery studies^34–36^. Participants were asked to complete the validated BREAST-Q questionnaire before surgery and at 3 and 12 months after surgery either electronically or on paper as per patient preference.

### Outcome measures

#### Patient preferences for oncoplastic breast-conserving surgery *versus* mastectomy and rates of successful breast-conserving surgery

The proportion of women who chose OPBCS when offered the procedure to avoid mastectomy and the proportion who underwent successful BCS after one or more procedures were calculated. Successful BCS was defined as clear tumour excision margins according to local guidelines. The management of women in whom excision was considered incomplete was explored, including the type and total number of additional surgical procedures required to achieve clear margins and the final surgical procedure.

#### Clinical outcomes

The total number of participants experiencing any breast surgery-related complication and a major complication, defined as requiring readmission or reoperation at both 3 and 12 months, was explored. The time to adjuvant treatment, defined as the time in days from the last oncological surgical procedure to the first dose of chemotherapy or fraction of radiotherapy, was calculated. The proportion of participants readmitted for complications, in whom further surgery to improve the appearance of the breast and/or reconstruction and/or achieve symmetry was either planned or had been performed by 12 months, was determined.

#### Patient-reported outcomes

Participants completed the BREAST-Q questionnaire before surgery and at 3 and 12 months after surgery. The BREAST-Q questionnaire is a validated measure robustly developed for patients undergoing breast surgery^37^. It has a modular design with multiple independently operating scales that explore satisfaction and quality of life. The core scales include ‘satisfaction with breasts’, ‘psychosocial well-being’, ‘physical well-being: chest’, and ‘sexual well-being’. The total score for each scale is calculated and Rasch transformed to generate a score out of 100, with higher scores indicating better outcomes. A change of four points (3 points for the ‘physical well-being: chest’ scale) is considered the minimum clinically important difference^38^.

### Sample size and statistical analysis

#### Sample size considerations

This was a feasibility study that aimed to explore the preferences and outcomes in a cohort of women offered OPBCS to avoid mastectomy. No formal sample size calculation was therefore undertaken, but recruitment of approximately 50 participants in each of the five groups of interest (TM, CWPF, simple mastectomy, IBR with implants, and free flaps) was planned to allow estimation of the distributions of outcomes after each procedure type to inform the sample size of a future definitive study.

#### Statistical analysis

Simple summary statistics were calculated to describe demographic, surgical, oncological, and outcome data per participant group and the cohort overall. Categorical data are presented as *n* (%) and continuous data are presented as median (interquartile range), range. Kruskal–Wallis and chi-squared tests were used to compare procedure groups for continuous and categorical variables respectively.

Groups were defined according to the first surgical procedure performed for baseline variables and according to the final procedure performed for postoperative variables and outcomes. Participants were categorized into four groups for the purposes of the analysis: TM, CWPF, simple mastectomy, and mastectomy **+** IBR. Women having IBR were analysed as one group due to the small number of patients who chose this option.

Questionnaires were scored according to the developers’ instructions and BREAST-Q scores for each main scale were compared across groups at baseline, 3 months, and 12 months using the Kruskal–Wallis test. Unadjusted 3- and 12-month scores for each BREAST-Q scale were compared with baseline scores in each group using the sign test. Then, 3- and 12-month scores were adjusted for baseline using linear regression and compared across procedure groups. Both analyses were repeated according to the initial procedure performed as a sensitivity analysis.

## Results

**Fig. 1:**
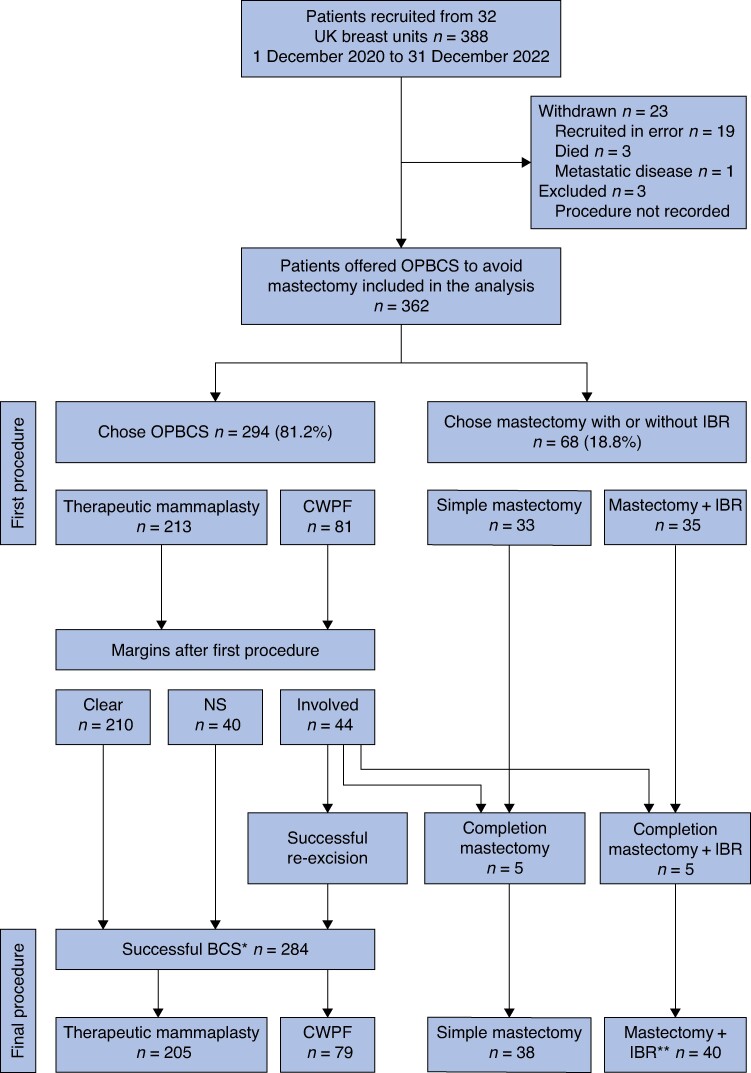
ANTHEM participant recruitment, decision-making, and outcomes *Includes 47 women assumed to have had successful oncoplastic breast-conserving surgery; margin status after index or re-excision surgery not explicitly stated. **Immediate breast reconstruction includes implant-based (19 women), free flap (17 women), and pedicled flap (4 women) procedures. OPBCS, oncoplastic breast-conserving surgery; IBR, immediate breast reconstruction; CWPF, chest wall perforator flap; NS, not stated.

Between 1 December 2020 and 31 December 2022, a total of 388 patients from 32 UK centres were recruited. Recruitment was extended from 12 to 25 months due to COVID-19-related delays in site opening and limited local research capacity. Of the 388 patients, 23 patients were withdrawn from the study (19 patients were recruited in error as they did not meet the inclusion criteria, 3 patients died, and 1 patient developed metastatic disease) and the records for 3 patients did not include details of the procedure performed and so these patients were excluded. A total of 362 participants were included in the present analysis (*Fig. 1*).

### Patient preferences for oncoplastic breast-conserving surgery *versus* mastectomy and rates of successful breast-conserving surgery

Of the 362 women offered OPBCS as an alternative to mastectomy, 294 (81.2%) women chose BCS with either a TM procedure (213 women) or a CWPF procedure (81 women). Of the 68 (18.8%) women electing to undergo mastectomy, over half (35 women) also chose to undergo IBR, with an implant-based (17 women, 48.6%), free flap (15 women, 42.9%), or pedicled flap (3 women, 8.6%) procedure (*Fig. 1*). Women choosing mastectomy + IBR were younger (*P* = 0.005) and more likely to have presented via the symptomatic pathway (*P* = 0.017), whereas women electing to undergo TM had higher BMIs than women in the other procedure groups. Bilateral surgery was more commonly performed in women having TM or mastectomy + IBR (*P* < 0.001) compared with the other groups and mastectomy with or without IBR was more likely to be performed in women with a multifocal disease at presentation (*P* = 0.004). There were no other significant differences between the procedure groups (*Table 1*).

**Table 1:** ANTHEM cohort demographics by the first surgical procedure performed

	Therapeutic mammaplasty, *n* = 213	Chest wall perforator flaps, *n* = 81	Simple mastectomy, *n* = 33	Mastectomy + IBR*, *n* = 35	*P*†
Age (years), median (i.q.r), range	58 (51–66), 27–83	58 (51–67), 23–80	61 (52–67), 33–86	53 (45–59), 31–66	0.005‡
**Age group**					
<=45 years	22 (10.3)	13 (16.1)	4 (12.1)	8 (22.9)	0.061
46–60 years	93 (43.7)	31 (38.3)	12 (36.4)	20 (57.1)	
>60 years	98 (46.0)	37 (45.7)	17 (51.5)	7 (20.0)	
**Presentation**					
Screening	111 (52.1)	31 (38.3)	14 (42.4)	10 (28.6)	0.017
Symptomatic	99 (46.5)	50 (61.7)	18 (54.6)	25 (71.4)	
Not reported	3 (1.4)	0 (0.0)	1 (3.0)	0 (0.0)	
BMI (kg/m^2^), median (i.q.r.), range	28.7 (25.4–34.6), 20.1–52.6	25.8 (23.7–28.1), 18.3–39.3	26.9 (24.6–30.0), 21.3–43.5	27.0 (25.6–31.1), 22.2–39.3	<0.001‡
**BMI group**					
Underweight (<18.5 kg/m^2^)	0 (0.0)	1 (1.2)	0 (0.0)	0 (0.0)	0.003
Normal (18.5–24.9 kg/m^2^)	46 (21.6)	28 (34.6)	10 (30.3)	6 (17.1)	
Overweight (25.0–29.9 kg/m^2^)	68 (31.9)	32 (39.5)	12 (36.4)	18 (51.4)	
Obese (>=30 kg/m^2^)	92 (43.2)	14 (17.3)	8 (24.2)	10 (28.6)	
Not reported	7 (3.3)	6 (7.4)	3 (9.1)	1 (2.9)	
Co-morbidities	105 (49.3)	32 (39.5)	16 (48.5)	14 (40.0)	0.314
Current/recent smoker	60 (28.2)	18 (22.2)	9 (27.3)	6 (17.1)	0.414
Neoadjuvant chemotherapy	31 (14.6)	8 (9.9)	8 (24.2)	9 (25.7)	0.073
**Focality**					
Unifocal	124 (58.2)	46 (56.8)	14 (42.4)	8 (22.9)	0.004
Multifocal	77 (36.2)	32 (39.5)	15 (45.5)	21 (60.0)	
Not reported	12 (5.6)	3 (3.7)	4 (12.1)	6 (17.1)	
**Preoperative nodal status**					
Negative	154 (72.3)	49 (60.5)	18 (54.6)	21 (60.0)	0.012
Positive	22 (10.3)	19 (23.5)	8 (24.2)	5 (14.3)	
Not reported	37 (17.4)	13 (16.0)	7 (21.2)	9 (25.7)	
**ASA grade**					
I	65 (30.5)	34 (42.0)	15 (45.5)	17 (48.6)	0.081
II	124 (58.2)	44 (54.3)	13 (39.4)	15 (42.9)	
III	17 (8.0)	2 (2.5)	2 (6.1)	1 (2.9)	
Not reported	7 (3.3)	1 (1.2)	3 (9.1)	2 (5.7)	
Bilateral surgery performed	81 (38.0)	4 (4.9)	3 (9.1)	9 (25.7)	<0.001
**Axillary surgery performed**					
None	41 (19.3)	10 (12.3)	1 (3.0)	0 (0.0)	0.009
Sentinel node biopsy/sample	125 (58.7)	49 (60.5)	22 (66.7)	26 (74.3)	
Axillary node clearance	17 (8.0)	9 (11.1)	6 (18.2)	6 (17.1)	
Not reported	30 (14.1)	13 (16.1)	4 (12.1)	3 (8.6)	

Values are *n* (%) unless otherwise indicated. *Immediate breast reconstruction includes implant-based (17 women), free flap (15 women), and pedicled flap (3 women) procedures. †Chi-squared test unless otherwise stated. ‡Kruskal–Wallis test. IBR, immediate breast reconstruction; i.q.r., interquartile range.

#### Adequacy of excision after oncoplastic breast-conserving surgery and rates of successful breast conservation

Adequacy of surgical excision was explicitly reported in 254 of 294 (86.4%) women opting for OPBCS. Of these, 210 of 254 (82.7%) women had clear surgical margins according to local guidelines after their index procedure and a further 27 of 44 (61.3%) women had clear margins after one (24 women) or more (3 women) successful attempts at re-excision. Overall, therefore, 237 of 254 (93.9%) women opting for OPBCS to avoid mastectomy had successful BCS.

Of the remainder, 10 of 254 (3.9%) women underwent completion mastectomy with (5 women) or without (5 women) IBR either as their second operation (4 women, 40.0%) or after one or more attempts at margin re-excision (6 women, 60.0%). Overall, in the 44 women requiring re-excision, 28 (63.6%) women required one additional operation to achieve clear margins and 9 (20.5%) women required two or more additional operations to achieve clear margins. The final surgical procedure was not specifically stated for 7 of 44 (15.9%) women. No differences were seen in the final surgical histology or the recommendations for adjuvant chemotherapy or endocrine treatment across the procedure groups. Adjuvant radiotherapy was recommended for 256 of 284 (90.1%) women undergoing successful OPBCS compared with 39 of 78 (50.0%) women having mastectomy with or without IBR (*P* < 0.001) (*Table 2*).

**Table 2:** Three-month clinical outcomes and multidisciplinary team decision-making by the final procedure performed*

Short-term clinical outcomes	Therapeutic mammaplasty, *n* = 205	Chest wall perforator flap, *n* = 79	Simple mastectomy, *n* = 38	Mastectomy + IBR†, *n* = 40	*P*‡
Any complication	57 (27.8)	16 (20.3)	11 (28.9)	12 (30.0)	0.546
Major complications requiring readmission/reoperation	10 (4.9)	2 (2.5)	0 (0.0)	10 (25.0)	<0.001
Total number of postoperative clinic visits, median (i.q.r.), range	3 (2–5), 0–21	3 (2–5), 0–11	3 (2–4), 0–16	4 (2–6), 1–15	0.190§
**Postoperative histology**					
T category					
Tis	47 (22.9)	12 (15.2)	6 (15.8)	6 (15.0)	0.524
T1	55 (26.8)	22 (27.8)	6 (15.8)	13 (32.5)	
T2	59 (28.8)	31 (39.2)	13 (34.2)	12 (30.0)	
T3	12 (5.9)	3 (3.8)	3 (7.9)	4 (10.0)	
pCR (post-NACT)	11 (5.4)	3 (3.8)	4 (10.5)	2 (5.0)	
Not reported	21 (10.2)	8 (10.1)	6 (15.8)	3 (7.5)	
N category					
pN0	84 (41.0)	33 (41.8)	11 (28.9)	16 (40.0)	0.462
pN1	30 (14.6)	14 (17.7)	11 (28.9)	11 (27.5)	
pN2	11 (5.4)	7 (8.9)	2 (5.3)	3 (7.5)	
pN3	4 (2.0)	1 (1.3)	2 (5.3)	1 (2.5)	
No axillary staging performed	39 (19.0)	10 (12.7)	2 (5.3)	1 (2.5)	
Not reported	37 (18.0)	14 (17.7)	10 (26.3)	8 (20.0)	
**MDT treatment recommendations**					
Chemotherapy					
Recommended	45 (22.0)	23 (29.1)	14 (36.8)	11 (27.5)	0.379
Not recommended	131 (63.9)	46 (58.2)	19 (50.0)	22 (55.0)	
Already received	24 (11.7)	7 (8.9)	4 (10.5)	7 (17.5)	
Not reported	5 (2.4)	3 (3.8)	1 (2.6)	0 (0.0)	
Radiotherapy					
Recommended	182 (88.8)	74 (93.7)	20 (52.6)	19 (47.5)	<0.001
Not recommended	19 (9.3)	3 (3.8)	17 (44.7)	21 (52.5)	
Not reported	4 (2.0)	2 (2.5)	1 (2.6)	0 (0.0)	
Endocrine therapy					
Recommended	148 (72.2)	56 (70.9)	20 (52.6)	28 (70.0)	0.118
Not recommended	53 (25.9)	22 (27.8)	17 (44.7)	11 (27.5)	
Not reported	4 (2.0)	1 (1.3)	1 (2.6)	1 (2.5)	
Time to adjuvant treatment (days), median (i.q.r.), range	64 (53–84), 12–235	63 (48–84), 8–272	77 (46–93), 39–108	72 (58–111), 20–139	0.356§

Values are *n* (%) unless otherwise indicated. *Includes ten women with unsuccessful OPBCS (TM, 8 women; and CWPF, 2 women) whose final procedure was simple mastectomy (5 women) or IBR (5 women). †Immediate breast reconstruction includes implant-based (19 women), free flap (17 women), and pedicled flap (4 women) procedures. ‡Chi-squared test unless otherwise stated. §Kruskal–Wallis test. IBR, immediate breast reconstruction; i.q.r., interquartile range; NACT, neoadjuvant chemotherapy; MDT, multidisciplinary team.

### Clinical outcomes at 3 and 12 months

The proportions of women experiencing postoperative complications at 3 months are summarized in *Table 2*. There were no differences in the rate of any complications or in the total number of postoperative clinic visits between groups, but women whose final procedure was an IBR experienced a significantly more major complications at 3 months than those having successful OPBCS or simple mastectomy (*P* < 0.001). No differences, however, were seen in the time to adjuvant treatment between the groups (*Table 2*).

Clinical outcomes at 12 months are summarized in *Table 3*. By 12 months, women in the IBR group were significantly more likely to require readmission for a complication related to their breast surgery than those in the other groups and those having mastectomy with or without IBR were significantly more likely to have undergone further surgery to improve the appearance of their chest and/or reconstruction or have it planned than those who had successful OPBCS. Few women had undergone or were awaiting symmetrization surgery at 12 months (*Table 3*).

**Table 3:** Twelve-month clinical outcomes by final procedure performed

	Therapeutic mammaplasty, *n* = 205	Chest wall perforator flap, *n* = 79	Simple mastectomy, *n* = 38	Mastectomy + IBR, *n* = 40	P*
Readmission for complications at 12 months	2 (1.0)	2 (2.5)	2 (5.3)	5 (12.5)	0.002
**Further surgery at 12 months to improve appearance of breast or breast reconstruction**	9 (4.4)	2 (2.5)	7 (18.4)	9 (22.5)	<0.001
Performed	6 (2.9)	2 (2.5)	3 (7.9)	5 (12.5)	<0.001
Planned	3 (1.5)	0 (0.0)	4 (10.5)	4 (10.0)	
**Type of surgery**					
Delayed breast reconstruction	0 (0.0)	0 (0.0)	4 (10.5)	2† (5.0)	0.175
Excision of dog ear	1 (0.5)	0 (0.0.)	1 (2.6)	0 (0.0)	
Removal of implant	0 (0.0)	0 (0.0)	0 (0.0)	2 (5.0)	
Lipofilling	4 (2.0)	2 (2.5)	0 (0.0)	3 (7.5)	
Risk-reducing mastectomy with or without IBR	0 (0.0)	0 (0.0)	1 (2.6)	1 (2.5)	
Other revision	2 (1.0)	0 (0.0)	1 (2.6)	0 (0.0)	
Symmetrization surgery to contralateral breast (planned or performed)	16 (7.8)	1 (1.3)	0 (0.0)	3 (7.5)	0.063

Values are *n* (%). *Chi-squared test. †Two patients having immediate implant-based reconstruction experienced implant loss and were awaiting delayed reconstruction. IBR, immediate breast reconstruction.

### Patient-reported outcomes

**Fig. 2:**
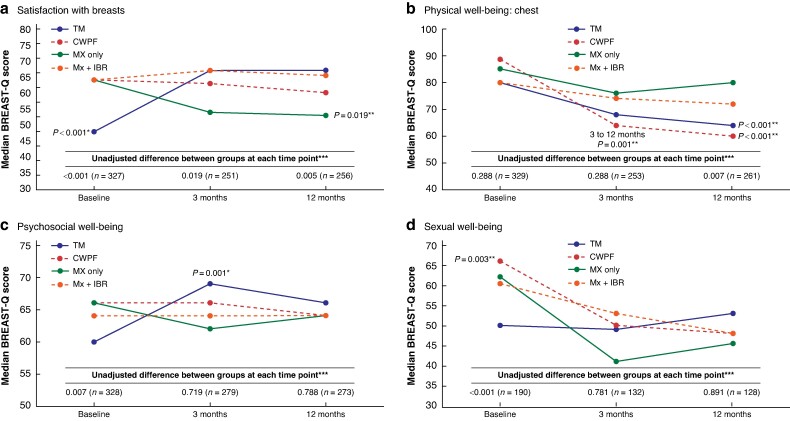
Changes in unadjusted BREAST-Q scores over time by the final procedure performed *Significant increase from baseline (sign test). **Significant decrease from baseline (sign test). ***Kruskal–Wallis test. TM, therapeutic mammaplasty; CWPF, chest wall perforator flap; Mx, mastectomy; IBR, immediate breast reconstruction.

At least one BREAST-Q scale was completed by 329 (90.9%), 279 (77.1%), and 273 (75.4%) women at baseline, 3 months, and 12 months respectively. Unadjusted BREAST-Q scores for each time point by the final procedure performed are shown in *Fig. 2* and detailed in *Table S1*. Although their baseline scores were the lowest overall, women in the TM group reported clinically meaningful and statistically significant increases in both the ‘satisfaction with breasts’ and ‘psychosocial well-being’ scores from baseline to 3 months that were maintained at 12 months. In contrast, ‘satisfaction with breasts’ decreased from baseline to 3 months in the mastectomy only group, with no improvement at 12 months. Both OPBCS groups (TM and CWPF) reported significant decreases in ‘physical well-being: chest’ scores from baseline to 3 months, with further decreases in scores between 3 and 12 months in the CWPF group (*Fig. 2*). Women undergoing CWPF also reported significant decreases in their ‘sexual well-being’ at 3 and 12 months, but the numbers of women completing this scale at each time point was relatively small. No other significant changes were seen (*Fig. 2*). These findings did not change when the analysis was repeated according to the initial procedure performed (data not shown).

When 3- and 12-month BREAST-Q scores were adjusted for baseline and compared with scores reported by women having mastectomy, those undergoing a successful TM procedure reported significantly higher ‘satisfaction with breasts’ scores at both 3 and 12 months and higher ‘psychosocial well-being’ scores at 12 months. Women having CWPF reported significantly higher ‘satisfaction with breasts’ than women in the mastectomy only group at 3 months, but not at 12 months. There were no significant differences in ‘satisfaction with breasts’ scores between the IBR and mastectomy only groups at either time point (*Table 4*). After adjusting for baseline, the decreases in ‘physical well-being: chest’ after OPBCS were less marked when compared with the mastectomy group and were only significant at 12 months, particularly in the CWPF group. No other significant differences between groups were seen at either time point (*Table 4*).

**Table 4:** Between group differences in mean Breast Q-scores at 3 and 12 months by the final procedure performed, adjusted for baseline scores

	Three-month difference (95% c.i.)	*P*	Twelve-month difference (95% c.i.)	*P*
**Satisfaction with breasts**	*n* = 240		*n* = 241	
Mastectomy only	Reference		Reference	
Therapeutic mammaplasty	12.9 (3.7,22.0)	0.006	15.9 (7.1,24.7)	<0.001
Chest wall perforator flaps	11.2 (1.3,21.1)	0.027	8.8 (−0.7,18.2)	0.069
Immediate breast reconstruction	7.8 (−5.2,20.8)	0.242	7.3 (−4.1,18.8)	0.209
**Physical well-being: chest**	*n* = 242		*n* = 246	
Mastectomy only	Reference		Reference	
Therapeutic mammaplasty	−3.6 (−10.5,3.2)	0.299	−8.8 (−16.7,−0.8)	0.030
Chest wall perforator flaps	−7.5 (−15.0,0.1)	0.052	−14.5 (−23.2,−5.8)	0.001
Immediate breast reconstruction	0.1 (−9.5,9.7)	0.988	−3.9 (−14.4,6.5)	0.458
**Psychosocial well-being**	*n* = 267		*n* = 256	
Mastectomy only	Reference		Reference	
Therapeutic mammaplasty	6.9 (−0.8,14.6)	0.079	8.4 (0.6,16.2)	0.034
Chest wall perforator flaps	0.9 (−7.5,9.3)	0.828	3.7 (−4.8,12.1)	0.393
Immediate breast reconstruction	−3.2 (−13.5,7.1)	0.543	3.8 (−6.1,13.8)	0.449
**Sexual well-being**	*n* = 117		*n* = 103	
Mastectomy only	Reference		Reference	
Therapeutic mammaplasty	6.4 (−7.9,20.8)	0.376	2.9 (−11.3,15.9)	0.738
Chest wall perforator flaps	3.3 (−12.5,19.1)	0.677	−3.5 (−18.7,11.6)	0.644
Immediate breast reconstruction	4.8 (−13.3,23.0)	0.600	−0.7 (−18.3,16.8)	0.933

## Discussion

This multicentre prospective cohort study exploring the outcomes of women offered OPBCS as an alternative to mastectomy demonstrates that, when given a choice between OPBCS and mastectomy, most women elect for OPBCS and, in over 90%, breast conservation is successful. Major complication rates at both 3 and 12 months were much lower after OPBCS than after mastectomy + IBR and women electing to have OPBCS required significantly fewer additional procedures to improve the appearance of their breast/chest wall than women having mastectomy with or without IBR. Women who had a successful TM procedure reported significant increases in their baseline ‘satisfaction with breasts’ and ‘psychosocial well-being’ scores at 3 and 12 months, highlighting a positive impact on their quality of life, particularly compared with those having a simple mastectomy. Women having OPBCS, however, reported worse ‘physical well-being: chest’ scores, particularly after CWPF. This is also seen after standard BCS and may reflect the impact of radiotherapy in this group^39^. Overall, these findings suggest that OPBCS should be offered as an alternative to mastectomy in all women who may be technically suitable for the procedure.

This multicentre prospective study is, to the authors knowledge, the first to directly compare preferences and outcomes in a cohort of women offered OPBCS to avoid mastectomy and to provide much needed evidence regarding the clinical and patient-reported outcomes of volume replacement and volume displacement techniques in this setting^40^. Consistent with previous studies^22,23,28,36,41^, this work confirms high rates of successful BCS and low rates of major complications after OPBCS, with few patients requiring revision surgery over time. It provides further evidence to support the beneficial impact of TM on women’s satisfaction and well-being^24,25^ and highlights better outcomes compared with patients having mastectomy. In this study, comparable benefits are not seen in either the CWPF group or the IBR group, which is somewhat unanticipated. One explanation may be that TM procedures combine removing the cancer with lifting/reducing the breast, potentially leaving women with an improvement in their appearance after treatment, whereas CWPF and IBR procedures aim to restore existing breast contour. Maintenance of baseline scores in these groups over time, therefore, may reflect a successful outcome for these women. Further work involving more patients and longer follow-up is now needed to explore whether and how the outcomes of different procedures continue to change over time, particularly after radiotherapy, to support women to make fully informed decisions about their surgical options.

This study has generated informative data, but there are limitations that require consideration. Firstly, study eligibility was based on the operating surgeon’s assessment that OPBCS was offered to avoid mastectomy. It is acknowledged that this assessment is highly subjective and that the high proportion of T1/2 cancers in the cohort is somewhat surprising. Notably, however, over 40% of women had surgery for multifocal/multicentric cancer, so T category alone may not accurately represent the extent of disease in the breast. In addition, the similarity in T category between the OPBCS and mastectomy groups suggests that these are comparable.

Study eligibility criteria mandated that women should be offered both OPBCS and mastectomy; these did not have to be offered equally and surgeon preference may have impacted decision-making. Given that participants were recruited from 32 centres with an interest and expertise in OPBCS, it is likely that this option was presented positively to patients and qualitative interviews with study participants highlighted that surgeons’ confidence in OPBCS was fundamental to women choosing the procedure^32^. This may mean that OPBCS is less acceptable as an alternative to mastectomy in units with less experience or that patients may not be offered potentially appropriate options due to lack of local expertise^31^. Similarly, the low complication and high success rates seen in these expert centres may not reflect the outcomes of OPBCS more widely. The similarity of these findings and previously published work^22,28,36^, however, suggests this is unlikely.

Very few women chose mastectomy with or without IBR in the study. This precluded more in-depth statistical analysis, including adjusting for confounding variables, such as BMI, receipt of bilateral surgery, and age, that may have impacted the findings. Small patient numbers also necessitated a combined analysis of patients in the IBR group and prevented the outcomes of individual reconstruction types from being explored. It is well established that the patient-reported outcomes of implant-based and free flap reconstruction differ^42,43^ and this combined analysis, together with the small sample size, may explain the unanticipated similarity in BREAST-Q scores between the mastectomy only and mastectomy + IBR groups. In addition, participants were only followed up for 12 months after surgery. Longer follow-up with embedded qualitative work is needed, particularly to explore the impact of radiotherapy on satisfaction and functional outcomes, as these effects are likely to become more apparent over time. Further work is needed to explore patient-reported outcomes in a larger, more definitive cohort, but the findings of this work significantly contribute to the growing body of evidence that supports surgeons offering OPBCS as an alternative to mastectomy.

There remains a need for high-quality research including long-term clinical, oncological, and patient-reported outcomes of OPBCS, to guide practice and support informed decision-making. This is an established national and international research priority^40,44,45^. Such studies should ideally be large-scale prospective international efforts designed in collaboration with patient advocates that will rapidly recruit large numbers of patients, producing high-quality generalizable data that will support surgeons and patients to make better decisions about their surgical options. OPBCS is likely to be a better option than mastectomy for many women, but the outcomes of volume replacement and volume displacement techniques differ. Patients with breast cancer are individuals, so should be fully informed about all relevant options, so that they can be supported to choose the procedure that best matches their goals and preferences. Further evidence is now needed to support and change practice.

## Supplementary Material

znae306_Supplementary_Data

## Data Availability

Data will be made available upon reasonable request from the corresponding author once analysis is complete.
