# Supplementary material for: Clinical and patient-reported outcomes in women offered oncoplastic breast-conserving surgery as an alternative to mastectomy: ANTHEM multicentre prospective cohort study
Source: Br J Surg. 2024 Dec 24;112(1):znae306. doi: 10.1093/bjs/znae306 (PMC11668256; doi:10.1093/bjs/znae306)
Supplement: znae306_Supplementary_Data [file znae306_supplementary_data.docx]

**Title: Clinical and patient-reported outcomes in women offered oncoplastic breast conserving surgery as an alternative to mastectomy: the ANTHEM multicentre, prospective cohort study**

Authors: Charlotte Davies^1^, Leigh Johnson^2^, Carmel Conefrey^2^, Nicola Mills^2^, Patricia Fairbrother^3^, Chris Holcombe^4^, Lisa Whisker^5^, William Hollingworth^2^, Joanna Skillman^6^, Paul White^7^, Douglas Macmillan^5^, Charles Comins^8^, Shelley Potter^1,9^

^1^Bristol Surgical and Perioperative Care Complex Intervention Collaboration, Translational Health Sciences, Bristol Medical School, University of Bristol, Learning and Research Building, Southmead Hospital, Southmead Road, Bristol, BS10 5NB,UK

^2^Population Health Sciences, Bristol Medical School, Canynge Hall, 39 Whatley Road, Bristol, BS8 2PS, UK ^3^Independent Cancer Patients Voice (ICPV)

^4^Linda McCartney Centre, Royal Liverpool and Broadgreen University Hospital, Prescot Street, Liverpool, L7 8XP, UK, ^5^Nottingham Breast Institute, Nottingham University Hospitals NHS Trust, Hucknall Road, Nottingham, NG5 1PB, UK

^6^Department of Plastic Surgery, University Hospitals Coventry and Warwickshire NHS Trust, Clifford Bridge Road, Coventry, CV2 2DX, UK

^7^Applied Statistics Group, University of the West of England, Bristol, UK

^8^University Hospitals Bristol Foundation NHS Trust

^9^Bristol Breast Care Centre, Southmead Hospital, Southmead Road, Westbury-on-Trym, Bristol, BS10 5NB, UK.

**Corresponding author.** Professor Shelley Potter

Translational Health Sciences, Bristol Medical School, University of Bristol, Learning and Research Building, Southmead Hospital, Southmead Road, Bristol, BS10 5NB, UK

Email: [Shelley.potter@bristol.ac.uk](mailto:Shelley.potter@bristol.ac.uk)

**Twitter** @drshelleypotter

**ORCID ID:** 0000-0002-6977-312X

**Supplementary Materials - Index**

| **Supplementary Results** |  |
| --- | --- |
| Supplementary Table 1: Unadjusted BREAST-Q scores by participant group | *page 2* |

**Supplementary Results**

**Supplementary Table 1: Unadjusted BREAST-Q scores by participant group**

|  | **Therapeutic mammaplasty** | | **Chest wall perforator flap** | | **Simple mastectomy** | | **Mastectomy and IBR** | |
| --- | --- | --- | --- | --- | --- | --- | --- | --- |
| **Satisfaction with Breasts** | n | Median  (95% CI) | n | Median  (95% CI) | n | Median  (95% CI) | n | Median  (95% CI) |
| Baseline | 185 | 48 (45-51) | 71 | 64 (60-68) | 34 | 64 (58-70) | 37 | 64 (59-69) |
| 3 months | 150 | 67 (64-70) | 59 | 63 (58-68) | 24 | 54 (46-62) | 18 | 67 (57-77) |
| 12 months | 144 | 67 (64-70) | 62 | 60 (55-65) | 26 | 53 (47-59) | 24 | 65.5 (58-73) |
|  |  |  |  |  |  |  |  |  |
| **Physical Well-being: Chest** | n | Median  (95% CI) | n | Median  (95% CI) | n | Median  (95% CI) | n | Median  (95% CI) |
| Baseline | 186 | 80 (77-83) | 72 | 88.5 (82-93) | 34 | 85 (78-92) | 37 | 80 (75-85) |
| 3 months | 149 | 68 (65-71) | 59 | 64 (60-68) | 25 | 76 (70-82) | 20 | 74 (66-82) |
| 12 months | 146 | 64 (61-67) | 62 | 60 (55-65) | 27 | 80 (72-88) | 28 | 72 (66-78) |
|  |  |  |  |  |  |  |  |  |
| **Psychosocial Well-being** | n | Median  (95% CI) | n | Median  (95% CI) | n | Median  (95% CI) | n | Median  (95% CI) |
| Baseline | 186 | 60 (57-63) | 71 | 66 (61-71) | 34 | 66 (59-73) | 37 | 64 (57-71) |
| 3 months | 163 | 69 (66-72) | 63 | 66 (62-70) | 28 | 62 (55-69) | 25 | 64 (55-73) |
| 12 months | 149 | 66 (63-69) | 64 | 64 (59-69) | 30 | 64 (57-71) | 30 | 64 (56-72) |
|  |  |  |  |  |  |  |  |  |
| **Sexual Well-being** | n | Median  (95% CI) | n | Median  (95% CI) | n | Median  (95% CI) | n | Median  (95% CI) |
| Baseline | 105 | 50 (46-54) | 37 | 66 (60-72) | 24 | 62 (53-71) | 24 | 60.5 (53-68) |
| 3 months | 72 | 49 (43-55) | 33 | 50 (41-59) | 12 | 41 (23-59) | 15 | 42 (42-64) |
| 12 months | 64 | 53 (46-60) | 32 | 48 (40-56) | 16 | 45.5 (37-54) | 16 | 48 (33-63) |

CI – confidence interval
